# Combination of clinical, radiomic, and “delta” radiomic features in survival prediction of metastatic gastroesophageal adenocarcinoma

**DOI:** 10.3389/fonc.2023.892393

**Published:** 2023-08-14

**Authors:** Satheesh Krishna, Andrew Sertic, Zhihui (Amy) Liu, Zijin Liu, Gail E. Darling, Jonathon Yeung, Rebecca Wong, Eric X. Chen, Sangeetha Kalimuthu, Michael J. Allen, Chihiro Suzuki, Elan Panov, Lucy X. Ma, Yvonne Bach, Raymond W. Jang, Carol J. Swallow, Savtaj Brar, Elena Elimova, Patrick Veit-Haibach

**Affiliations:** ^1^ Department of Medical Imaging, University of Toronto, Toronto, ON, Canada; ^2^ Department of Biostatistics, Princess Margaret Cancer Centre, University Health Network, Toronto, ON, Canada; ^3^ Division of Thoracic Oncology, Toronto General Hospital, University Health Network, Toronto, ON, Canada; ^4^ Division of Radiation Oncology, Princess Margaret Hospital, University Health Network, Toronto, ON, Canada; ^5^ Division of Medical Oncology, Princess Margaret Cancer Centre, University Health Network, Toronto, ON, Canada; ^6^ Division of Pathology, Toronto General Hospital, University Health Network, Toronto, ON, Canada; ^7^ Department of Surgery, Princess Margaret Cancer Centre, University Health Network, Toronto, ON, Canada

**Keywords:** gastric, esophageal, carcinoma, radiomics, survival

## Abstract

**Objectives:**

To identify combined clinical, radiomic, and delta-radiomic features in metastatic gastroesophageal adenocarcinomas (GEAs) that may predict survival outcomes.

**Methods:**

A total of 166 patients with metastatic GEAs on palliative chemotherapy with baseline and treatment/follow-up (8–12 weeks) contrast-enhanced CT were retrospectively identified. Demographic and clinical data were collected. Three-dimensional whole-lesional radiomic analysis was performed on the treatment/follow-up scans. “Delta” radiomic features were calculated based on the change in radiomic parameters compared to the baseline. The univariable analysis (UVA) Cox proportional hazards model was used to select clinical variables predictive of overall survival (OS) and progression-free survival (PFS) (p-value <0.05). The radiomic and “delta” features were then assessed in a multivariable analysis (MVA) Cox model in combination with clinical features identified on UVA. Features with a p-value <0.01 in the MVA models were selected to assess their pairwise correlation. Only non-highly correlated features (Pearson’s correlation coefficient <0.7) were included in the final model. Leave-one-out cross-validation method was used, and the 1-year area under the receiver operating characteristic curve (AUC) was calculated for PFS and OS.

**Results:**

Of the 166 patients (median age of 59.8 years), 114 (69%) were male, 139 (84%) were non-Asian, and 147 (89%) had an Eastern Cooperative Oncology Group (ECOG) performance status of 0–1. The median PFS and OS on treatment were 3.6 months (95% CI 2.86, 4.63) and 9 months (95% CI 7.49, 11.04), respectively. On UVA, the number of chemotherapy cycles and number of lesions at the end of treatment were associated with both PFS and OS (p < 0.001). ECOG status was associated with OS (p = 0.0063), but not PFS (p = 0.054). Of the delta-radiomic features, delta conventional HUmin, delta gray-level zone length matrix (GLZLM) GLNU, and delta GLZLM LGZE were incorporated into the model for PFS, and delta shape compacity was incorporated in the model for OS. Of the treatment/follow-up radiomic features, shape compacity and neighborhood gray-level dependence matrix (NGLDM) contrast were used in both models. The combined 1-year AUC (Kaplan–Meier estimator) was 0.82 and 0.81 for PFS and OS, respectively.

**Conclusions:**

A combination of clinical, radiomics, and delta-radiomic features may predict PFS and OS in GEAs with reasonable accuracy.

## Introduction

Gastroesophageal cancer is one of the leading causes of cancer deaths worldwide. The incidence of adenocarcinomas has significantly increased in Western countries over the last four decades, partly attributable to the rise in obesity and gastroesophageal reflux disease (1). Up to 40% of patients present with stage IV disease (2). While different therapy options currently appear on the horizon, chemotherapy is currently the mainstay of treatment in metastatic gastroesophageal adenocarcinomas (GEAs). However, the prognosis remains poor with a 1-year and 5-year survival of 30% and 5%, respectively, in stage IV GEAs (3). To make informed treatment choices that match patients’ preferences and goals, information regarding treatment outcomes in terms of survival is necessary. Current prediction models in GEAs for prognosis rely mostly on clinical parameters and have been found to be limited (4, 5).

Medical imaging is routinely used to monitor and/or predict treatment response for cancer treatment. This is based on subjective analysis of the imaging appearance of the tumor on the baseline CT (size, invasiveness, and Heterogeneity) and treatment/follow-up CT after 8–12 weeks of chemotherapy (change in size, change in heterogeneity, and enhancement) (6). Subjective visual assessment of CT scans is limited in providing prognostic information due to the limitation of visual interrogation and interobserver variability. Radiomics converts medical images (CT scans) into a set of high dimensional, mineable, and quantitative features (like shape, texture, and transformation), which provide tumor information that is not easily identifiable by simple visual analysis (7). CT-derived radiomic analysis provides biomarkers that have shown promise in correlation with tumor biology and aggressiveness, which in turn predict survival (8). While previous studies have utilized radiomics in the evaluation of treatment response in neoadjuvant settings in patients with resectable locally advanced GEAs, there is a lack of literature in the palliative setting in metastatic GEAs (9, 10).

In addition to providing information on internal tumor heterogeneity, radiomic evaluation on the baseline and treatment/follow-up CT can provide information regarding tumor response following chemotherapy. This “delta” radiomic measures change in radiomic information between the baseline and treatment/follow-up CT and can detect spatial response variations (11). The relative net change of radiomic features in longitudinal images may potentially identify and quantify early therapy-induced changes and thereby predict survival (11). To our knowledge, the utility of delta-radiomics in metastatic GEAs has not been previously assessed.

Given the importance of all the mentioned variables in prognosis, i.e., 1) clinical features and 2) baseline radiomic features (as a biomarker of tumor heterogeneity and aggressiveness) and 3) delta-radiomic features (as a biomarker of tumor response to chemotherapy), it is desirable to have a model combining all these variables to predict survival. The aim of this study is to identify a combined clinical, radiomic, and delta-radiomic model that may be predictive of overall survival and progression-free survival in metastatic GEAs.

## Methods

This retrospective study was approved by the local research ethics board, and the need for informed consent was waived. Patients with pathologically proven metastatic gastroesophageal adenocarcinoma in our tertiary referral center who received palliative chemotherapy between 2009 and 2020 were identified from an institutional registry retrospectively. All patients underwent a “baseline” CT (within 2 months prior to the start of chemotherapy) and a “treatment/follow-up” scan after 8–12 weeks of chemotherapy. Patients without a contrast-enhanced “baseline” or “treatment/follow-up” portal venous phase CT were excluded. Overall, 166 patients were identified. Clinical and pathology data were obtained including patient and tumor characteristics, treatment, and follow-up data, such as Eastern Cooperative Oncology Group (ECOG) status at clinical presentation, history of smoking and alcohol use, and clinical and pathological staging (according to the 8th edition of the American Joint Committee on Cancer (AJCC)).

### Image analysis

All patients received iodinated intravenous contrast media-enhanced CT scan in the portal venous phase. All CT scans were performed on 64 or 320 multidetector scanners (Aquilion 64 or Aquilion One, Canon (formerly Toshiba) Medical Systems Corporation, Otawara, Japan). Images were acquired using 120 kVp, gantry rotation speed of 0.5–0.75 s, and automatic mAs setting with an accepted noise level standard deviation of 13–15 Hounsfield units. The volumetric acquisition was reconstructed at a 5-mm slice thickness every 2.5 mm in the axial plane and a 3-mm thickness every 3 mm in the coronal and sagittal planes. The manufacturer’s standard soft tissue filter (FC04) was used for the reconstruction. Intravenous contrast consisted of 100 cc of iopromide (Ultravist 370; Bayer Healthcare, Berlin, Germany) injected at 3 cc/s. The abdomen was then imaged in the portal venous phase, 70 s after initiation of contrast bolus injection. The scan acquisition and scan parameters were stable throughout the study period.

**Figure 1:**
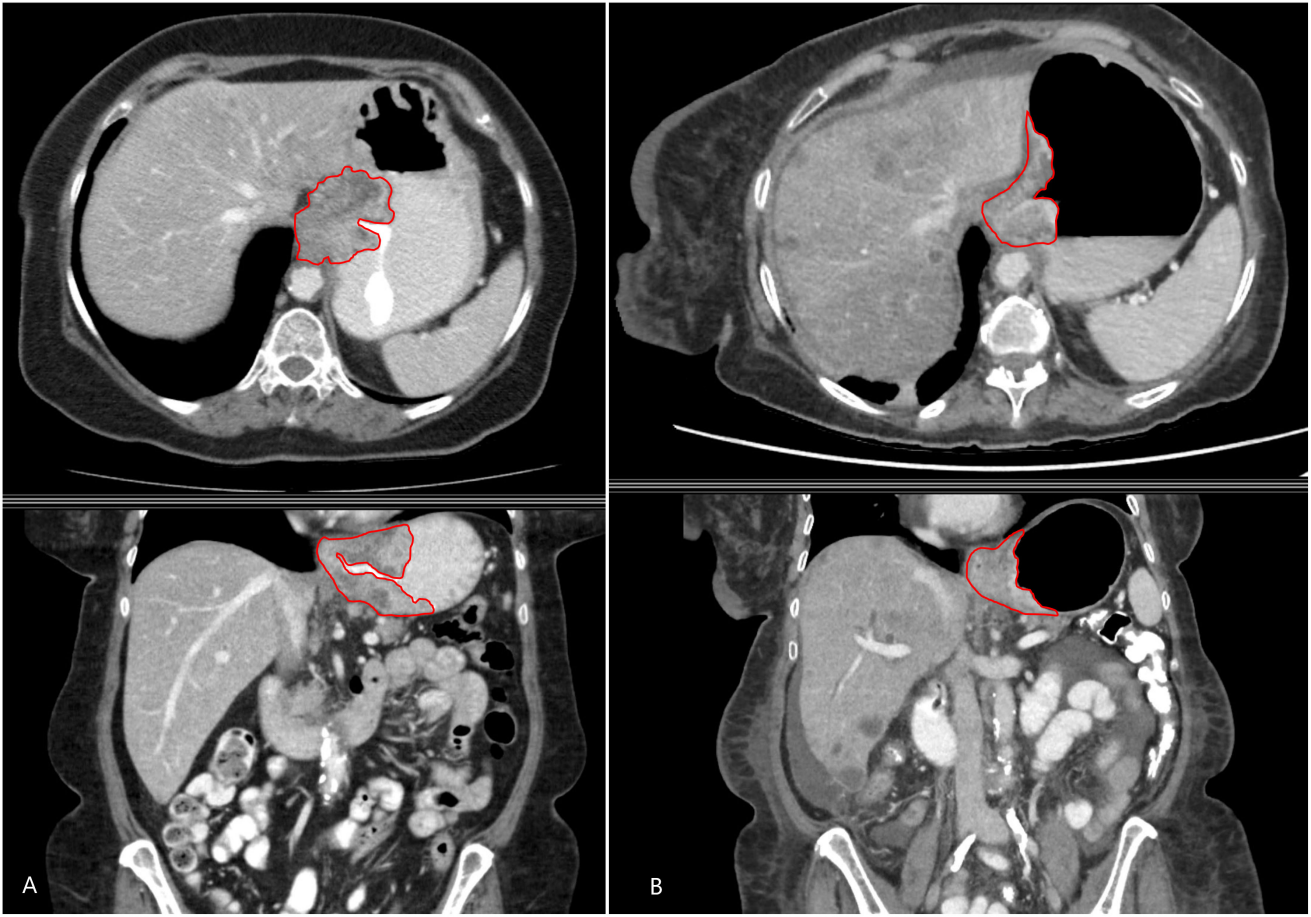
A 48-year-old man with metastatic gastroesophageal adenocarcinoma. Axial (above) and coronal (below) contrast-enhanced CT images showing the primary gastroesophageal adenocarcinoma and the contours of segmentation on the **(A)** baseline and **(B)** treatment/follow-up (8–12 weeks on chemotherapy) CT.

The digital imaging and communication medical data (400-bit grayscale) of the CT scans were retrieved from the image archiving system. Feature extraction software (LIFEx v3.74, CEA-SHFJ, Orsay, France) was used for manual region-of-interest three-dimensional segmentation and texture analyses. All segmentations and feature extraction of the tumor were performed by one author (AS) with 5 years of experience with gastrointestinal imaging (**Figure 1**). All segmentation was three-dimensional and was performed manually slice by slice to cover the entire tumor lesion. A total of 76 subdivided texture features were extracted, including conventional indices (including, but not limited to, minimum, maximum, average, and standard deviation values), discretized indices, first-order histogram-based parameters, shape-derived parameters and texture features from second or higher order [gray-level co-occurrence matrix (GLCM), gray-level zone length matrix (GLZLM), neighborhood gray-level dependence matrix (NGLDM), and gray-level run length matrix (GLRLM)]. Radiomic features were extracted from the segmented regions of interest (ROIs) in both the baseline and treatment/follow-up images.

### Statistical analysis

Summary statistics were used to describe patient, disease, and treatment characteristics. The Kaplan–Meier (KM) method was used to estimate overall survival (OS) and progression-free survival (PFS) and their 95% confidence interval (CI). Univariable Cox proportional hazards models were fitted to select clinical variables predictive of OS and PFS with a p-value <0.05 indicating significance. Multivariable Cox proportional hazards models were fitted to lesion-level data, with robust standard errors via clustering to account for intra-patient correlation.

To develop the final multivariable model, first, significant clinical variables with a p-value <0.05 were selected from the univariable analyses (UVAs). Then, the radiomic features and “delta” features (defined as change between baseline and treatment/follow-up scans) were assessed one by one in multivariable analyses (MVAs) in the presence of the selected clinical variables. Radiomic features with a p-value <0.01 (here a more stringent threshold to account for the larger number of radiomic features than clinical features) were selected to further assess their pairwise correlation. Only non-highly correlated features (Pearson’s correlation coefficient <0.7) were included in the final model.

To validate the performance of the models, leave-one-out cross-validation at the lesion level was conducted. First, one lesion was removed and used as validation, and a Cox model with robust standard errors was trained in the remaining data. Then, the trained model was used to predict the outcome of the validation lesion. These steps were repeated for each lesion in turn. The predicted outcomes of the lesions from all patients were combined to calculate the receiver operating characteristics (ROC) curve and the 1-year area under the ROC curve (AUC) with the Kaplan–Meier estimator using the *survivalROC* package in R version 4.0.2.

## Results

Of the 166 patients included in the study, 114 (69%) were male and 52 (31%) were female with a median age of 59.8 years (26.9, 80.7). Of the patients, 84% were non-Asian (n = 139). The majority of patients were ECOG 0 or 1 (32% and 57%, respectively), and 11% had ECOG 2 or above. The baseline patient characteristics are shown in **Table 1**.

**Table 1:** Clinical characteristics of patients included in the study.

Characteristics	N = 166
**Age (years),** Median (Min, Max)	59.8 (26.9, 80.7)
Sex	
Female	52 (31)
Male	114 (69)
Race	
Asian	27 (16)
Non-Asian	139 (84)
**Height (m)**, Median (Min, Max)	1.7 (1.3, 2)
**Weight (kg)**, Median (Min, Max)	70.8 (38.7, 111)
**BMI (kg/m^2^)**, Median (Min, Max)	24.4 (15.7, 47.4)
Alcohol	
Frequent/daily	22 (13)
Occasional	75 (45)
Past	17 (10)
Smoking	
Current	22 (13)
Ex-smoker	55 (33)
ECOG	
0	53 (32)
1	94 (57)
2	16 (10)
3	3 (2)

BMI, body mass index; ECOG, Eastern Cooperative Oncology Group.

**Figure 2:**
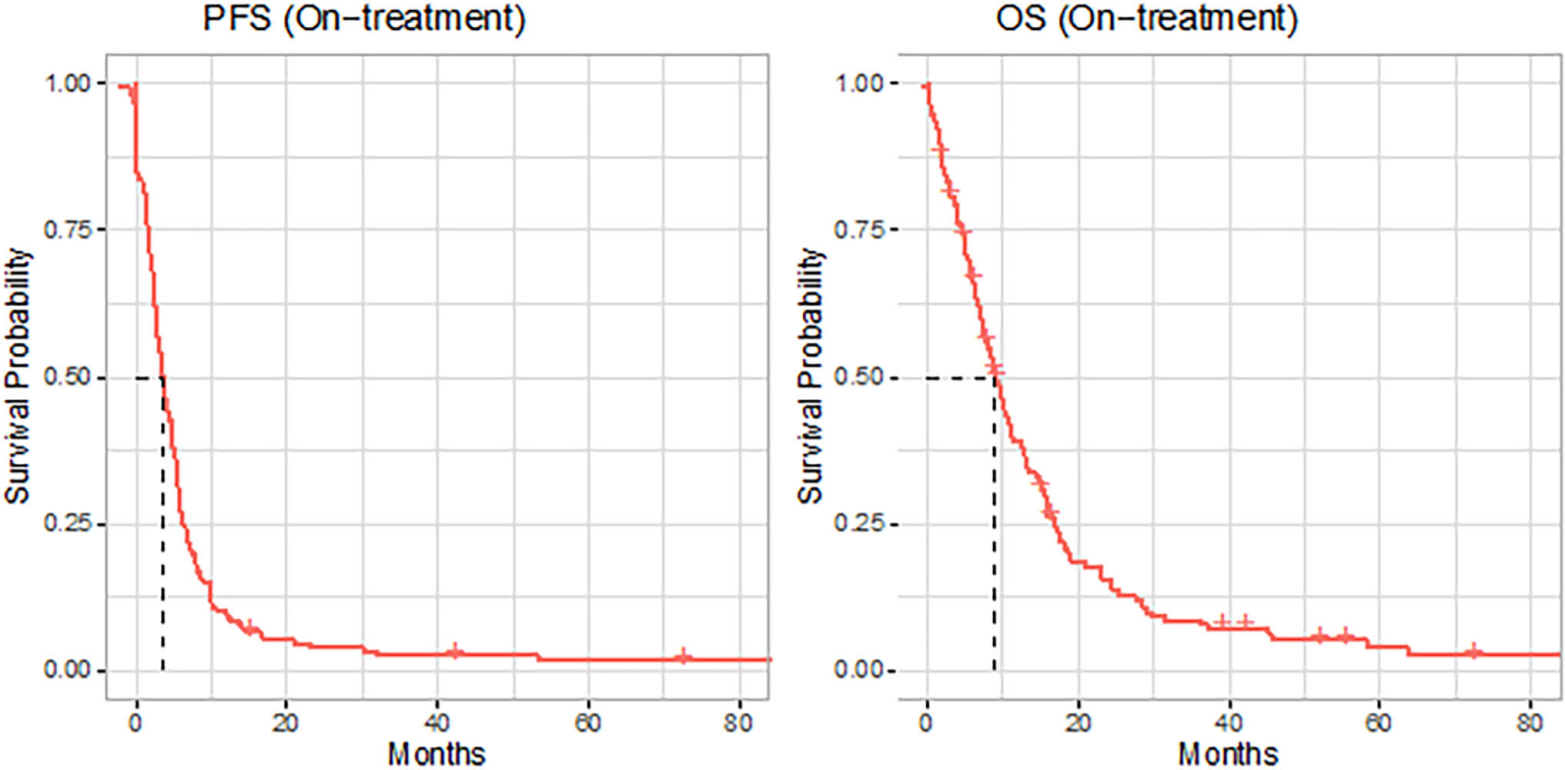
Kaplan–Meier (KM) curves (red solid lines) for progression-free survival (PFS) and overall survival (OS) of the full cohort, with dashed line indicating the median.

All patients had metastatic gastroesophageal adenocarcinoma. Of the patients, 117 (70%) had locoregional lymph node metastasis, and 69 (42%) had distant lymph node metastasis. The other common sites of metastasis were the liver (n = 63, 38%), peritoneum (n = 56, 34%), bone (n = 31, 19%), and brain (n = 5, 3%). Information regarding the sites of metastasis and the number of lesions at “baseline” and on the “treatment/follow-up” scans is summarized in **Table 2**. The median PFS and OS on treatment were 3.6 months (95% CI 2.86, 4.63) and 9 months, respectively (95% CI 7.49, 11.04) (**Figure 2**).

**Table 2:** Details of metastasis and chemotherapy cycles of patients included in the study.

Characteristics	n = 166
Sites of metastasis	
**Lymph node (locoregional)**	117 (70)
**Distant lymph node**	69 (42)
**Liver**	63 (38)
**Peritoneal**	56 (34)
**Bone**	31 (19)
**Brain**	5 (3)
**Other**	54 (33)
**Number of chemo cycles**, Median (Min, Max)	6 (1, 42)
Number of lesions at baseline	
1	52 (31)
2	31 (19)
3	44 (27)
4	18 (11)
5	20 (12)
6	1 (1)
Number of lesions after treatment	
None	5 (3)
1	52 (32)
2	28 (17)
3	36 (22)
4	22 (14)
5	23 (14)

On UVA, the number of chemotherapy cycles and the number of lesions on the follow-up scans were associated with both PFS and OS (p < 0.001). ECOG status at baseline was associated with OS (p = 0.006), but not PFS (p = 0.054). Similarly, the presence of brain metastasis was adversely associated with OS (p < 0.001) and not PFS (p = 0.078). The rest of the clinical variables were not associated with either OS or PFS (p > 0.05). The UVA of clinical variables for PFS and OS is summarized in **Supplementary Tables 1**, **2**.

An MVA Cox proportional hazards model was developed incorporating the clinical features, radiomic features, and the “delta” features for PFS and OS. Both these models incorporated features from all three categories (clinical, baseline radiomic, and delta features). Of the radiomic features, shape compacity and NGLDM contrast were used in both models (for OS and PFS). Of the delta features, delta conventional HUmin, delta GLZLM GLNU, and delta GLZLM LGZE were incorporated into the model for PFS, while delta shape compacity was incorporated in the model for OS. The final models used for PFS and OS including the radiomic variables are summarized in **Tables 3**, **4**.

**Table 3:** Multivariable Cox proportional hazards model for progression-free survival.

Covariate	HR (95%CI)	p-Value
**Number of chemo cycles**	0.88 (0.81, 0.95)	**0.001**
**Number of lesions after treatment**	1.17 (1, 1.38)	0.051
**GLZLM ZLNU**	0.97 (0.86, 1.1)	0.670
**NGLDM contrast**	0.97 (0.95, 0.99)	**0.014**
**Delta conventional HUmin**	0.89 (0.77, 1.03)	0.110
**Delta GLZLM GLNU**	1.23 (1.06, 1.42)	**0.006**
**SHAPE compacity**	1.28 (1.14, 1.45)	**<0.001**
**Delta GLZLM LGZE**	1.06 (1.03, 1.09)	**<0.001**

GLZLM, grey-level zone length matrix; ZLNU, zone length non-uniformity; NGLDM, neighborhood grey-level dependence matrix; HU, Hounsfield units; GLNU, gray-level non-uniformity; LGZE, low gray-level zone emphasis.Values in bold p<0.05.

**Table 4:** Multivariable Cox proportional hazards model for overall survival.

Covariate	HR (95%CI)	p-Value
**ECOG**	1.73 (1.3, 2.3)	**<0.001**
**Brain metastasis (*vs.* no)**	7.66 (4.49, 13.07)	**<0.001**
**Number of chemo cycles**	0.89 (0.85, 0.92)	**<0.001**
**Number of lesions after treatment**	1.38 (1.21, 1.58)	**<0.001**
**Shape compacity**	1.09 (0.98, 1.22)	0.1
**GLZLM size**	0.84 (0.75, 0.95)	**0.003**
**Shape surface (mm^2^)**	1.15 (1.02, 1.29)	**0.019**
**Delta shape compacity**	1.33 (1.07, 1.64)	**0.009**
**NGLDM contrast**	1.04 (1.01, 1.07)	**0.017**

ECOG, Eastern Cooperative Oncology Group; GLZLM, grey-level zone length matrix; NGLDM, neighborhood grey-level dependence matrix.Values in bold p<0.05.

**Figure 3:**
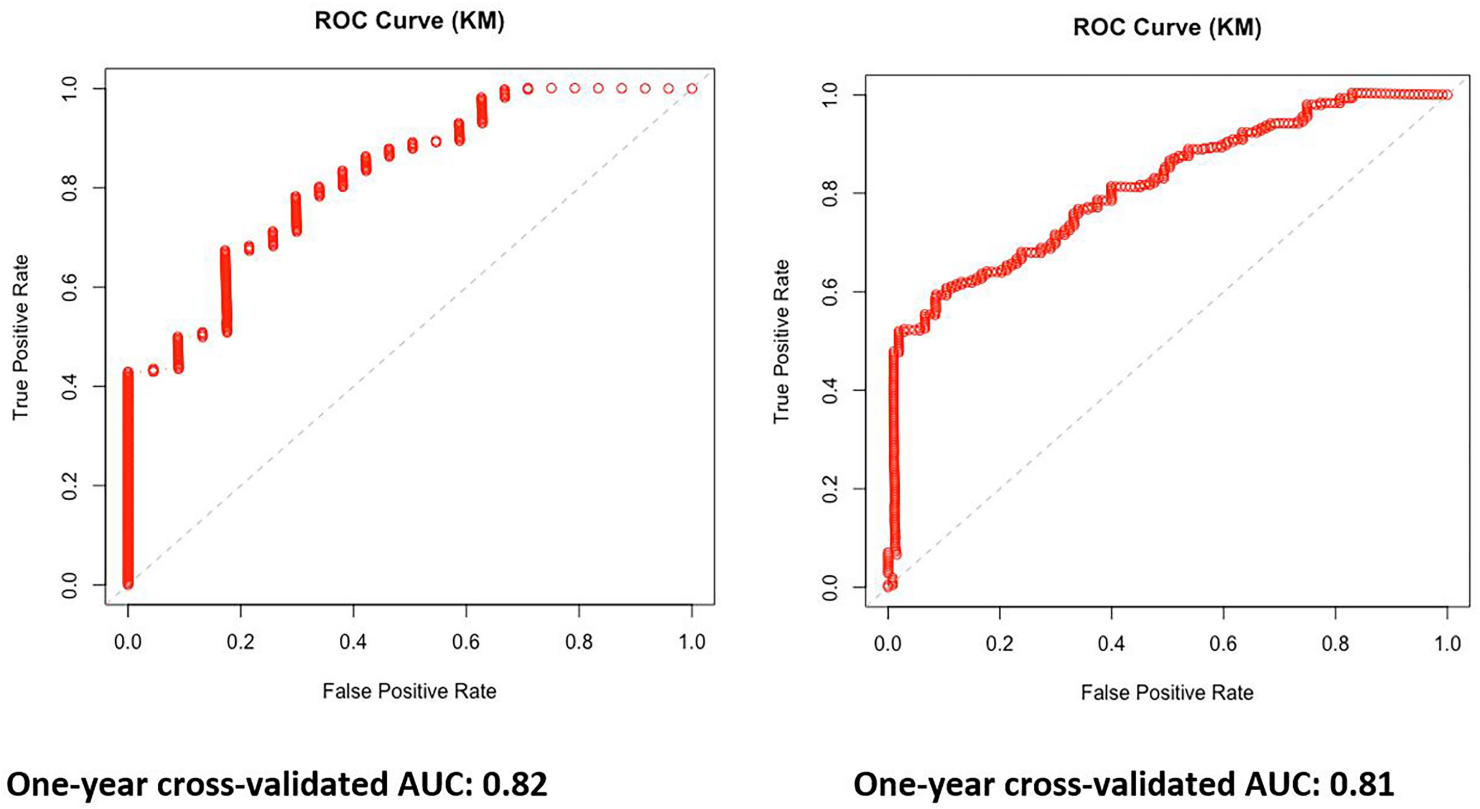
One-year area under the curve (AUC) Kaplan–Meier estimator curves based on the multivariable model for progression-free survival (PFS) and overall survival (OS).

The leave-one-out cross-validation method was used, and the 1-year AUC (Kaplan–Meier estimator) was calculated with the area under the curve of 0.82 and 0.81 for PFS and OS, respectively (**Figure 3**).

## Discussion

In our current study, we evaluated the value of combining patient-related clinical variables, with quantitative imaging variables in survival prediction in patients with metastatic GEAs. Specifically, we assessed the combined benefit of radiomic features, which are biomarkers of tumor aggressiveness, along with delta-radiomic features, which are biomarkers of tumor response to treatment. We found that a combined model incorporating variables from all three subsets (clinical, radiomics, and delta-radiomics) has incremental value in the prediction of survival.

Of the clinical variables, expectedly, higher ECOG status was associated with poor survival, which is consistent with prior literature (12). Higher tumor burden on the treatment/follow-up CT assessed by the number of residual metastatic lesions on the treatment/follow-up scan was also associated with both poor OS and PFS. It is already known that high tumor burden in the form of multiple sites of metastasis and the number of metastasis pre-treatment is associated with poor survival (13). This is consistent with emerging knowledge of higher survival in oligometastatic disease, with some preliminary studies encouraging aggressive local treatment of oligometastasis to reduce tumor burden to improve survival (12, 14). However, our study also shows that the number of metastatic lesions after 8–12 weeks of chemotherapy is important in the prediction of survival, which underlines the importance of follow-up imaging not only in terms of radiomic evaluation. In addition, presence of brain metastasis was shown to have poor OS but not PFS in our study. It has been previously shown that patients with gastroesophageal adenocarcinomas are more likely to have brain metastasis when compared with patients with esophageal squamous cell carcinomas (15). However, previous studies have not shown a difference in survival in patients with brain metastasis in GEAs (16). Finally, our study shows improved PFS and OS association with the number of cycles of chemotherapy, which is an obvious and expected finding.

Of the radiomic features, NGLDM contrast and shape compacity were used in both models. NGLDM contrast, which is the intensity difference between neighboring regions, has been identified as a marker for intratumoral heterogeneity and is associated with both PFS and OS (17). Shape compacity (Area^1.5/Volume) reflects how compact the volume is. The more tubular and elongated the mass is, the higher the compacity, which in our study was associated with worse PFS. This is concordant with prior studies showing that a longer length of tumor is associated with worse PFS (12). A similar association of compacity with PFS has been identified in gastric lymphoma (18). Similarly, a higher surface area of the tumor was associated with worse OS. In addition, high delta shape compacity was associated with poor OS. A high delta shape compacity would potentially indicate a tumor with worsening compacity compared to baseline, which would indicate an increase in tumor length or development of an infiltrative pattern on treatment. Thus, this is potentially a quantitative marker for worsening on treatment, which as expected was associated with worse OS. Delta-compacity has been shown to be significant in prior studies of advanced gastric cancer (19). GLZLM provides information on the size of homogeneous zones for each gray level in three dimensions. Delta GLZLM GLNU and delta GLZLM LGZE provide information on changes in heterogeneity between baseline and the treatment/follow-up CT and were associated with PFS. Loss of heterogeneity and improving homogeneity on the treatment/follow-up CT are known indicators of treatment response (20). Previously, delta GLZLM features have been shown to be useful in locally advanced hypopharyngeal cancers, and specifically, delta LGZE has been useful in head and neck cancers (21, 22). Thus, overall, radiomic features and delta-radiomic features predict survival by providing quantitative information regarding tumor characteristics and the change in tumor characteristics on treatment. A combination of these quantitative imaging biomarkers with clinical features was associated with survival in our study.

It needs to be pointed out there are no other publications currently available that evaluated “delta” radiomic correlations in the setting of palliative gastroesophageal cancer. Given the potential toxicity of chemotherapy, objective measurements of tumor response are important. Several publications and trials evaluated radiomic parameters with the ultimate goal of clinical routine implementation of radiomic evaluation. Since imaging is performed in a multitude of follow-up settings, follow-up radiomic evaluation and the clinical value thereof need to be investigated, too.

Our study has several limitations. Owing to the retrospective nature of the study cohort from a single center, selection bias cannot be entirely excluded. Given that the study cohort is relatively small, we did cross-validation for statistical robustness but no further validation of our results. In doing so, we would have had to split our study cohort into training and validation data sets, which would decrease the overall statistical robustness. Our results need to be validated externally preferably in prospective multicenter cohorts. This is especially important because radiomic parameters may be susceptible to alternate CT scanners, reconstruction algorithms, and imaging parameters, which may differ in other institutions. However, some of that variation was accounted for in this study already, as patients were imaged partly on different scanners. Direct clinical applicability is currently limited, as the tools to obtain radiomic parameters are not readily available on routine radiology PACS platforms and currently need separate workstations and platforms for image segmentation and feature extraction, however noting that automated radiomics evaluation tools are already on the horizon.

Radiomic analysis based on baseline and 8–12 weeks’ treatment/follow-up contrast-enhanced CT has the potential to predict survival in patients with metastatic GEAs treated with chemotherapy. A combination of clinical (patient/tumor-related features), radiomic (biomarker for tumor characteristics), and delta-radiomic (biomarker for tumor response to chemotherapy) features may predict survival in metastatic GEAs. These models require further prospective validation.

## Data availability statement

The original contributions presented in the study are included in the article/**Supplementary Material**. Further inquiries can be directed to the corresponding author.

## Ethics statement

The studies involving human participants were reviewed and approved by Research Ethics Board University Health Network. Written informed consent for participation was not required for this study in accordance with the national legislation and the institutional requirements.

## Author contributions

SKr – Conceptualization, Formal analysis, Investigation, Methodology. Roles/Writing – original draft. AS – Data curation. ZAL – Formal analysis. ZL – Formal analysis. GD – Validation, Writing – review & editing. JY– Validation, Writing – review & editing. RW – Supervision, Writing – review & editing. EC – Writing – review & editing. SKa – Writing – review & editing. MA – Data curation. CS – Data curation. EP – Data curation. LM – Writing – review & editing YB – Writing – review & editing. RJ – Writing – review & editing. CS – Writing – review & editing. SB – Writing – review & editing. EE – Supervision. PV-H – Conceptualization, Investigation, Methodology, Supervision, Roles/Writing – original draft, Writing – review & editing. All authors contributed to the article and approved the submitted version.
