# Supplementary material for: Combination of clinical, radiomic, and “delta” radiomic features in survival prediction of metastatic gastroesophageal adenocarcinoma
Source: Front Oncol. 2023 Aug 14;13:892393. doi: 10.3389/fonc.2023.892393 (PMC10461093; doi:10.3389/fonc.2023.892393)
Supplement: Supplementary file 1 [file Table_1.docx]

## Supplementary Table 1: Univariable Cox proportional hazards model for progression free survival

| **Covariate** | **HR (95%CI)** | **Global p-value** |
| --- | --- | --- |
| **Age** | 1.00 (0.99,1.01) | 0.97 |
| **Sex** | 0.98 (0.68,1.41) | 0.91 |
| **Race** | 0.71 (0.45,1.12) | 0.14 |
| **Height** | 0.9 (0.15,5.36) | 0.91 |
| **Weight** | 1.00 (0.99,1.01) | 0.49 |
| **BMI** | 0.98 (0.95,1.02) | 0.33 |
| **Alcohol** |  | 0.22 |
| **Smoking** |  | 0.54 |
| **ECOG** | 1.27 (1,1.61) | 0.054 |
| **Lymph node metastasis** | 1.11 (0.77,1.59) | 0.58 |
| **Distant metastasis** | 1.02 (0.72,1.43) | 0.93 |
| **Liver metastasis** | 1.22 (0.87,1.72) | 0.25 |
| **Peritoneal metastasis** | 1.27 (0.89,1.8) | 0.18 |
| **Bone metastasis** | 0.88 (0.57,1.37) | 0.58 |
| **Brain metastasis** | 2.25 (0.91,5.54) | 0.078 |
| **Other metastasis** | 1.01 (0.71,1.43) | 0.96 |
| **Number of chemo cycles** | 0.92 (0.88,0.96) | $<$**0.001** |
| **Number of lesions after treatment** | 1.20 (1.07,1.34) | **0.002** |

## Supplementary Table 2: Univariable Cox proportional hazards model for overall survival

| **Covariate** | **HR(95%CI)** | **Global p-value** |
| --- | --- | --- |
| **Age** | 1.00 (0.99,1.01) | 0.99 |
| **Sex** | 0.79 (0.54,1.16) | 0.22 |
| **Race** | 0.72 (0.44,1.17) | 0.18 |
| **Height** | 0.46 (0.07,2.93) | 0.41 |
| **Weight** | 0.99 (0.98,1) | 0.24 |
| **BMI** | 0.98 (0.94,1.02) | 0.30 |
| **Alcohol** |  | 0.59 |
| **Smoking** |  | 0.96 |
| **ECOG** | 1.44 (1.11,1.88) | **0.006** |
| **Lymph node metastasis** | 1.36 (0.93,1.98) | 0.11 |
| **Distant metastasis** | 1.19 (0.83,1.69) | 0.34 |
| **Liver metastasis** | 1.17 (0.82,1.67) | 0.38 |
| **Peritoneal metastasis** | 1.18 (0.82,1.7) | 0.38 |
| **Bone metastasis** | 1.22 (0.78,1.93) | 0.38 |
| **Brain metastasis** | 5.53 (2.15,14.25) | $<$**0.001** |
| **Other metastasis** | 0.89 (0.62,1.29) | 0.54 |
| **Number of chemo cycles** | 0.90 (0.85,0.95) | $<$**0.001** |
| **Number of lesions after treatment** | 1.28 (1.13,1.44) | $<$**0.001** |
